# Das „Mindset“ bei der Durchführung einer chirurgischen Atemwegssicherung durch AnästhesistInnen

**DOI:** 10.1007/s00101-026-01696-w

**Published:** 2026-06-02

**Authors:** Jan Carlo Del Tedesco, Lion Sieg, Markus Flentje, Vera Hagemann, Kai Johanning, Lars Friedrich, Hendrik Eismann

**Affiliations:** 1 Klinik für Anästhesiologie und Intensivmedizin, Medizinische Hochschule Hannover, Carl-Neuberg-Straße 1, 30625 Hannover, Deutschland; 2 Klinik für Anästhesiologie und Operative Intensivmedizin, Klinikum Lippe, Röntgenstraße 18, 32756 Detmold, Deutschland; 3 Fakultät für Wirtschaftswissenschaften, Universität Bremen, Enrique-Schmidt-Straße 1, 28359 Bremen, Deutschland; 4 Klinik für Anästhesiologie, operative Intensivmedizin, Notfallmedizin und Schmerztherapie, Klinikum Bielefeld, Teutoburger Straße 50, 33604 Bielefeld, Deutschland

**Keywords:** Koniotomie, Mentales Modell, Atemwegsmanagement, Crisis Ressource Management, Experteninterview, Cricothyroidotomy, Surgical airway management, Mental model, Crisis resource management, Expert interview

## Abstract

**Hintergrund:**

Die chirurgische Atemwegssicherung in der Situation des „cannot intubate – cannot oxygenate“ ist eine gefürchtete und multidimensional herausfordernde Situation für Teams in der Anästhesiologie und Notfallmedizin. Zahlreiche Faktoren können die Durchführung der Koniotomie beeinflussen. Neben der Erfahrung, dem Wissen und den praktischen Fähigkeiten scheinen hierbei auch das eingesetzte Material und das „Mindset“ des tätigen Personals eine zentrale Rolle zu spielen.

**Ziel der Arbeit:**

Ziel dieser Studie war die möglichst umfassende Darstellung von Erfahrungen, Einstellungen und Wissen im Hinblick auf die chirurgische Atemwegssicherung bei ärztlichen Mitarbeitenden einer Klinik für Anästhesiologie und Intensivmedizin. Die Studie folgte auf eine 2017 durchgeführte Datenerhebung zum Thema Koniotomie, auf deren Basis das klinikinterne Weiterbildungskonzept angepasst worden war.

**Methodik:**

Es handelt sich um eine gemischte qualitative und quantitative Studie. Abteilungsintern führten wir eine fragebogengeleitete Umfrage durch; nachfolgend luden wir ÄrztInnen in Weiterbildung, Fach- und OberärztInnen zu Experteninterviews ein.

**Ergebnis:**

Insgesamt konnten 59 Fragebögen von ärztlichen Mitarbeitenden der Klinik für Anästhesiologie und Intensivmedizin ausgewertet werden. Zudem führten wir insgesamt 4 Gruppeninterviews mit jeweils 6 bis 8 TeilnehmerInnen durch. Wir konnten zeigen, dass die Teilnahme an Skill- und Simulationstraining die subjektive Handlungskompetenz signifikant erhöht. Im Gesamtkollektiv bleibt die Sicherheit bei Entscheidung und Durchführung jedoch gering. In den Interviews zeigten sich weitere anästhesiologische Themenkomplexe, die das Thema chirurgische Atemwegssicherung indirekt betreffen und Einfluss auf das Atemwegsmanagement haben. So wurden die Rolle des Anästhesie-Ausweises beleuchtet und die Weiterbildung im Bereich fiberoptische Intubation aufgegriffen.

**Schlussfolgerung:**

Die Koniotomie im Rahmen des fehlgeschlagenen Atemwegsmanagements ist und bleibt eine der herausforderndsten Szenarien für anästhesiologische Teams. Regelmäßige Fortbildung als Skill-Training mit nachfolgender Einbettung in Simulationsszenarien können die subjektive Handlungskompetenz signifikant steigern. Die Unsicherheit über einen objektiv messbaren oder erhebbaren Punkt, der zweifelsfrei die Koniotomie nach sich zieht, bleibt weiterhin als hemmender Faktor bestehen und gilt es, in der weiteren Entwicklung von Trainingsprogrammen zu adressieren.

**Zusatzmaterial online:**

Die Online-Version dieses Beitrags (10.1007/s00101-026-01696-w) enthält den verwendeten Fragebogen. Bitte scannen Sie den QR-Code.

## Abstract (en)

**Background:**

The surgical establishment of a secure airway in a cannot intubate-cannot oxygenate situation represents a feared and multidimensionally challenging situation in anesthesiology and emergency medicine teams. Numerous factors can influence the performance of a cricothyroidotomy. In addition to experience, knowledge and practical skills, the materials used and the mindset of the personnel also appear to play a significant role.

**Aim of the study:**

This study aimed to provide a comprehensive picture of experiences, attitudes and knowledge on surgical airway management of medical personnel at a department of anesthesiology and intensive care medicine. This study builds on data collected on cricothyroidotomy in 2017, which changed the internal training concept adaptation of the hospital.

**Methodology:**

This study used a mixed qualitative and quantitative approach. We conducted a survey using questionnaires sent to all physicians of the department of anesthesiology, followed by expert interviews with doctors in training, specialists and senior physicians.

**Results:**

We could evaluate 59 questionnaires from the medical personnel. Additionally, we conducted 4 group interviews, each with 6–8 participants. We could demonstrate the significant influence of skill and simulation teaching programs on coniotomy; however, in the total collective the safety of decisions and performance remains low. Interviews covered a wide range of anesthesiological topics that indirectly affect and influence the topic of surgical airway management. The role of the anesthesia identification card was explained and the further education in the field of awake fiberoptic intubation was examined.

**Conclusion:**

Cricothyroidotomy in the context of failed airway management is and remains one of the most challenging scenarios for anesthesiology teams. Regular continuing education in the form of skills training, followed by integration into simulation scenarios, can significantly increase the subjective competence; however, the well-known uncertainty about an objectively measurable or detectable point that unequivocally indicates a cricothyroidotomy remains an inhibiting factor that needs to be addressed in the further development of training programs.

Die chirurgische Atemwegssicherung als Endstrecke des präklinischen wie klinischen Atemwegsalgorithmus stellt das behandelnde Team vor große Herausforderungen. Die geringe Inzidenz der Maßnahme, gepaart mit wenigen und oft unrealistischen Trainingsangeboten bei gleichzeitig maximaler Invasivität der Maßnahme, führt bei ÄrztInnen zu Unsicherheiten und häufig inkohärenten Einschätzungen der eigenen Handlungskompetenz. Ziel dieser Studie war es, die subjektive Perspektive der Behandelnden im Hinblick auf die Koniotomie und ihre Einbettung in das vielfältige Atemwegsmanagement strukturiert aufzuarbeiten und darzustellen.

## Einleitung

Die Atemwegssicherung und das Management des schwierigen Atemweges stellen eine zentrale Handlungskompetenz im Fachgebiet der Anästhesiologie dar. Endlich et al. konnten 2020 retrospektiv zeigen, dass etwa 56 % aller schwierigen Atemwege unerwartet auftraten und insbesondere Notfallpatienten betroffen waren [7]. Mittlerweile stehen viele Möglichkeiten zur Verfügung, um das Problem der Atemwegssicherung zu adressieren. Die Situation des „cannot intubate – cannot oxygenate“ (CICO) bleibt als gefürchtete Endstrecke des fehlgeschlagenen Atemwegsmanagements jedoch weiterhin bestehen. Etwa 52 % aller AnästhesistInnen berichteten von mindestens einer CICO-Situation in ihrem Berufsleben [26].

Ob die Koniotomie oder die Notfalltracheotomie das Verfahren der ersten Wahl zur chirurgischen Atemwegssicherung unter Zeitdruck darstellt, bleibt unklar. Nach Novellierungen der Leitlinie Atemwegsmanagement wird die Notfalltracheotomie durch einen „versierten Arzt“ [25], und nicht wie 2015 durch einen „versierten Chirurgen“ [24], mit exzellenter Routine empfohlen. Dennoch ist in den meisten Kliniken Konsens, dass die Koniotomie das Verfahren der Wahl zur chirurgischen Atemwegssicherung darstellt. Aufgrund der unsicheren Datenlage kann keine abschließende Entscheidung für oder gegen ein spezielles Koniotomie-Verfahren getroffen werden. Zentral ist jedoch eine frühzeitige Vorbereitung der Materialien. Letzteres soll Hemmschwellen reduzieren, aber auch durch regelmäßigen Sichtkontakt aktiv an eine mögliche Anwendung erinnern. Es zeigt sich, dass vielfach zu lange mit der Durchführung der chirurgischen Atemwegssicherung gewartet wird [2]. Der Fall Elaine Bromiley im Jahr 2005 steht hierfür fast archetypisch [1]. Die Klinik der Autoren lehrt aktuell die chirurgische Koniotomie-Technik mittels Bougie. Begründet wird dies mit einer relativ hohen Erfolgsrate [13, 15], der schnellen Anwendung [23] und dem raschen Erlernen [30]. Sämtliche Materialien zur Durchführung der Koniotomie finden sich auf allen Atemwegswagen und in den Notfallrucksäcken der Klinik.

Die chirurgische Atemwegssicherung bleibt eine der herausforderndsten Szenarien der anästhesiologischen Tätigkeit. Da dieses sehr invasive Verfahren selten angewandt wird, fordert es das Anästhesie-Personal als „High Responsibility Team“ ([11]; Tab. 1) sowohl fachlich als auch psychisch heraus. Eismann et al. zeigten, dass 69 % der befragten AnästhesistInnen nie eine Koniotomie durchgeführt haben und sich selbst als gering handlungskompetent einschätzten [5]. Der Weiterbildungsbedarf ist dementsprechend hoch. Studien weisen darauf hin, dass die stark schwankende Komplikationsrate (0–53 %) [17] von vielerlei Faktoren abhängig ist, nicht nur von Vorerkrankungen, sondern auch abhängig vom Setting [9] und dem Kompetenzniveau des eingesetzten Teams.

**Tab. 1:** Definition von „High Responsibility Teams“ nach Hagemann et al. [11]

Konsequenzen nach Fehlverhalten	Klassische Teams	High Responsibility Teams
Reversibilität der Ereignisse?	In der Regel ja	In der Regel nein
Körperliche und psychische Schäden?	Nein	Ja
Wem wird geschadet?	Dem Team und dem Unternehmen	Dem Team, dem Unternehmen und Dritten
Verantwortung für das Leben anderer?	Nein	Ja
Abbruch der Situation möglich?	Ja	Nein
Arbeitsunterbrechung möglich?	Pausen sind möglich	Pausen sind in der Regel nicht möglich
Mediendruck/Öffentlichkeit?	In der Regel nicht	Ja

Neben der Evaluation des neu etablierten Fortbildungsformates aus Skill- und Simulationstraining (Fokus Atemwegsmanagement, „HAINS Airway Kurs“) soll diese Studie Erfahrungen, Einstellungen und das Wissen der ÄrztInnen einer Universitätsklinik im Hinblick auf das Atemwegsmanagement erheben und einen besonderen Fokus auf die Koniotomie als Notfallmaßnahme legen. Dabei interessierten wir uns insbesondere für die Rolle des „Mindset“ als Summe der Denk- und Verhaltensmuster, der kognitiven und metakognitiven Konzepte, die das Anästhesie-Team zu entsprechenden Maßnahmen befähigen. Zudem sollten Herausforderungen, Chancen und Optimierungsmöglichkeiten herausgearbeitet werden, welche durch ihre Implementierung eine sichere, strukturierte und effiziente Anwendung des Atemwegsalgorithmus und der Koniotomie ermöglichen.

## Methoden

### Ethik

Diese gemischt qualitativ sowie quantitative Studie wurde durch die Ethikkommission der Medizinischen Hochschule (10892_BO-K-2023 vom 09.02.2023) positiv votiert.

### Quantitative Datenerhebung

Es wurde eine fragebogengestützte Umfrage im ärztlichen Team der Klinik für Anästhesiologie und Intensivmedizin durchgeführt. Inhaltlich griff dieser 2017 gestellte Fragen wieder auf, um zu einer ausreichenden Vergleichbarkeit zu gelangen. Teilnehmende waren zu beiden Zeitpunkten aktuelle MitarbeiterInnen der Klinik für Anästhesiologie und Intensivmedizin. Der Fragenpool verblieb identisch. Insbesondere die Zahl durchgeführter chirurgischer Atemwegssicherungen, die subjektive Handlungskompetenz und das präferierte Koniotomie-Verfahren erschienen relevant. Das Transferklima, definiert als Sammelbegriff für die generelle, insbesondere sozial vermittelte Umsetzbarkeit des Erlernten im beruflichen Setting, wurde mit einem von Hagemann et al. modifizierten und validierten Fragebogen erhoben [6, 10].

### Qualitative Datenerhebung

**Abb. 1:**
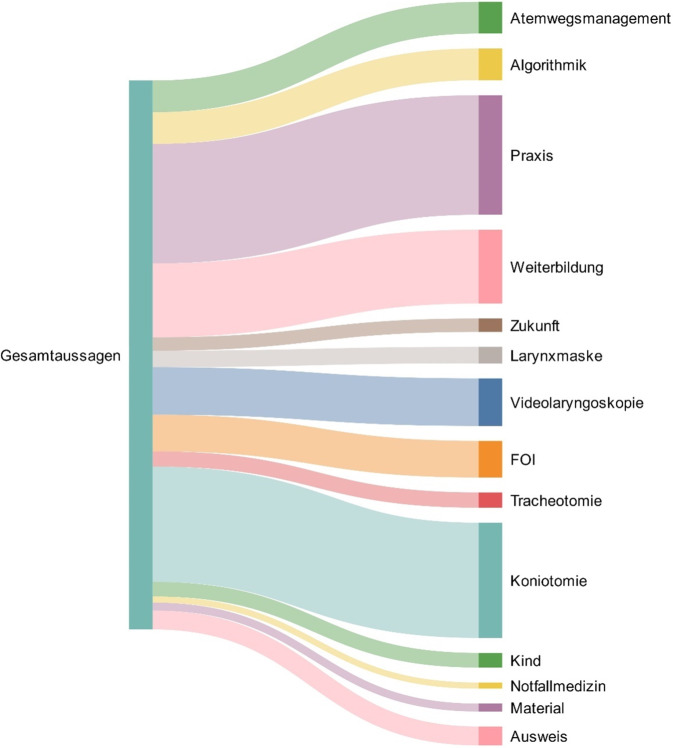
Sankey-Diagramm zur Zuordnung aufgezeichneter Aussagen in sämtlichen Fokusgruppeninterviews. Alle Aussagen „*n*“ sämtlicher Experteninterviews (*n* = 491) wurden eingeschlossen. Aussagen wurden teilweise mehreren Kategorien „k“ zugeordnet (k = 900)

Zur differenzierten Evaluation der Erfahrungen sowie Einstellungen der Teilnehmenden wurden nach der quantitativen Datenerhebung zwei Gruppeninterviews mit ÄrztInnen in Weiterbildung sowie zwei Gruppeninterviews mit Fach- sowie OberärztInnen durchgeführt. Die Teilnahme basierte auf Freiwilligkeit. Wir orientierten uns am Konzept der Experteninterviews nach Meuser und Nagel [19] und den Qualitätsstandards für qualitative Forschung (COREQ) [28]. Zunächst erhoben wir basale demographische Daten wie Dauer der klinischen Tätigkeit sowie Kompetenzniveau. Entlang von Trigger-Fragen sowie -Aussagen konnten die Teilnehmenden nachfolgend frei von ihren Erfahrungen berichten. Sämtliche Interviews wurden von einem ärztlichen Kollegen ohne klinikinterne Führungs- oder Leitungsaufgaben moderiert. Es existierte keine Verblindung zwischen ProbandInnen und Interviewer. In der Gruppe Gesprochenes wurde notiert und nicht audiovisuell aufgenommen, um die Compliance zur Teilnahme zu erhöhen. Die Notizen wurden nachfolgend, transkribiert und paraphrasiert, schließlich induktiv zu einzelnen Themenkomplexen wie z. B. „Algorithmik“ oder „Videolaryngoskopie“ zusammengefasst (Abb. 1). Die hier vorgelegte Zitierweise „>>“ und „<<“ dient der Abgrenzung zu einer Abschrift aus audiovisuellen Aufnahmen. Sie soll betonen, dass es sich hierbei um eine Rekonstruktion von schriftlichen Notizen handelt. Die Ergebnisse der qualitativen Datenerhebung sind exemplarisch in Tab. 2 dargestellt. Eine nähere Beschreibung erfolgt gemäß den „Standards for Reporting Qualitative Research“ [22] bereits mit Einordnung und Interpretation innerhalb der Diskussion.

**Tab. 2:** Darstellung relevanter Aussagen aus den Experteninterviews. „>>“ sowie „<<“ sollen den Transkriptionsprozess verdeutlichen, welcher sich diskret von audiovisuellen Aufnahmen unterscheidet [4].

Rolle der Koniotomie	
1	*>> Ja, die chirurgische Atemwegssicherung ist auch eine Kernkompetenz der Anästhesie, aber auf einem anderen Kompetenzlevel als die ITN. <<*
2	*>> Das notwendige Equipment ist sehr einfach, man braucht nicht viel für die Koniotomie. <<*
3	*>> An Material für eine chirurgische Atemwegssicherung kommt in meinem Kopf nur das „Fig. 11 – Skalpell“ und der Bougie vor. <<*
Hemmende Faktoren	
1	*>> Die Durchführung einer chirurgischen Atemwegssicherung hat mich vor allem Überwindung gekostet. <<*
2	*>> Die chirurgische Atemwegssicherung sollte anästhesiologische Kernkompetenz sein, irgendwie hat man das Problem ja auch ausgelöst. <<*
3	*>> Die Erfahrenen werden eher durch ihre Erfahrungen gehindert, wir AssistentInnen haben eher die Sorge, was vergessen zu haben. <<*
4	*>> Meine Hoffnung wäre, dass ich bei der Entscheidung zur Koniotomie nicht alleine bin, denn ich würde denken, dass es primär an mir liegt. <<*
5	*>> Meine persönliche Schwelle zur Koniotomie ist hoch, da ich denke, dass die frustrane Atemwegssicherung auch primär an mir liegen könnte. <<*
6	*>> Eigentlich müsste der Trigger eine unzureichende Maskenbeatmung, eine nichtpositionierbare Larynxmaske und eine frustrane Intubation mittels Videolaryngoskop sein. <<*
7	*>> Ein Herzfrequenzabfall wäre für mich ein Trigger für eine Koniotomie. Oder die Sättigung unter 50* *%, wenn klar ist, dass die Intubation nicht geht.<<*
Blick auf das Atemwegsmanagement und den Algorithmus	
1	*>> Der Atemwegsalgorithmus der Klinik ist schon idiotensicher, aber nicht wie SOAP‑M als Checkliste. <<*
2	*>> Ich habe das Gefühl, dass ich den Algorithmus durch Teaching erlernt habe und weniger über Papier. <<*
3	*>> Der Atemwegsalgorithmus der Klinik wirkt auf den ersten Blick wirklich unübersichtlich, die Eskalationsstufen wendet man intuitiv an. <<*
Weiterbildung und Koniotomie	
1	*>> Grundkenntnisse des Atemwegsmanagements müssen in den ersten Jahren vorhanden sein – bis zum ersten NEF-Dienst auf jeden Fall! <<*
2	*>> Nein, eine chirurgische Atemwegssicherung sollte nicht nur von einem Facharzt/einer Fachärztin oder einer höheren Position durchgeführt werden. Die KollegInnen sind ja mit drei Jahren Berufserfahrung auf dem NEF, dann gehört das auch dazu. <<*
Weitere relevante Themen	
1	*>> Es gibt keinen Nachteil, wenn man jeden PatientIn mit einem Videolaryngoskop intubiert. <<*
2	*>> Die Skills bei der Videolaryngoskopie sind besser, und es ist weniger Teamwork (auch mit dem Patienten) notwendig als bei der FOI, daher ist es einfacher. <<*
3	*>> Wenn der Mund nicht aufgeht, ist die FOI alternativlos. <<*
4	*>> Was ist mit einem Patienten/einer Patientin bei schwierigen Rahmenbedingungen wie z.* *B. ICU oder Katheter-Tisch? Wenn die Maskenbeatmung gut geht, ist dann kein Anästhesie-Ausweis nötig? <<*
5	*>> Zur Ausstellung eines Ausweises wird der Facharztstandard gefordert, aber oft wird nicht erneut durch den Facharzt/Fachärztin nachgeschaut. <<*
6	*>> Würden mehr Anästhesie-Ausweise unser Procedere ändern? Beim überraschenden 1:25.000-Fall wäre ein Ausweis gut. <<*

### Statistische Auswertung

Die statistische Analyse erfolgte in MS Excel (Microsoft®, Excel, 2023, Version 16.78, Redmond, WA, USA) und SPSS (IBM Corporation, 2023, Version 29.0; IBM, Armonk, NY, USA). Die Darstellung des Sankey-Diagrammes erfolgte mit der opensource Software SankeyMATIC.

Normalverteilung wurde mittels Shapiro-Wilk-Test überprüft. Korrelationen innerhalb der im Fragebogen erhobenen Parameter wurden mittels Spearman Rangkorrelation evaluiert. Mittels Mann-Whitney-U-Test wurden Unterschiede in den Gruppen von TeilnehmerInnen mit und ohne Fortbildung auf Signifikanzen getestet. Hierfür ergab sich nach Berechnung mittels G*Power ein *n* = 54 bei zweiseitiger Testung und erwartetem großem Effekt.

Ein Vergleich mit den Daten von Eismann et al. [5] gelang mittels Chi-Quadrat-Test und Mann-Whitney-U-Test. Sofern Voraussetzungen des Chi-Quadrat-Testes nicht erfüllt waren, wurde der Exakte Test nach Fisher verwendet.

## Ergebnisse

### Quantitative Datenerhebung

#### Rücklauf und Demographie

Insgesamt wurden 158 Mitarbeitende angeschrieben. Es konnten 59 von 61 rückläufigen Fragebogen ausgewertet werden. Dies entspricht bei 158 Fragebogen einer Rückläuferquote von 38,6 %. Zwei Fragebogen wurden aufgrund von Ablehnung der Datenschutzbestimmungen ausgeschlossen. Die Anzahl stellt die in der Literatur angegebene, durchschnittliche Nettorückläuferquote von Fragebogenstudien dar (ca. 30 %) [18]. Wir konnten ein ausgeglichenes Verhältnis im Hinblick auf das Geschlecht (44,1 % weiblich) und Kompetenzniveau (57,6 % ÄrztInnen in Weiterbildung) erzielen. 28,8 % der Teilnehmenden hatten 1 bis 2 Jahre Berufserfahrung sowie 16,9 % mehr als 20 Jahre Berufserfahrung. 30,5 % der Teilnehmenden waren 26 bis 30 Jahre alt sowie 37,3 % älter als 40 Jahre.

#### Chirurgische Atemwegssicherungen

Insgesamt konnten 27 Koniotomien durch Mitarbeitende der Klinik für Anästhesiologie und Intensivmedizin dokumentiert werden (*N* = 16,27 % der Befragten).

Die subjektive Handlungskompetenz liegt im Median bei 4 von 10 (1 = niedrigste mögliche subjektive Kompetenz, 10 = höchstmögliche subjektive Kompetenz), 72,8 % beurteilten ihre Fähigkeiten niedriger oder gleich 5. Je erfahrener die KollegInnen waren (Spearman’s ρ = 0,422, *p* < 0,001) oder je höher ihre Koniotomie-Erfahrung war (Spearman’s ρ = 0,406, *p* < 0,001), desto höher war ihre subjektive Handlungskompetenz.

#### Weiterbildung

Die Umsetzung der 2017 neu erarbeiteten Weiterbildungskonzepte (eintägiger Kurs mit einer Kombination aus Skill-Training und Simulation) führte trotz COVID-19-Pandemie zu einer signifikant höheren Beteiligung an Fortbildungsveranstaltungen. Insgesamt haben 64,4 % der Befragten an einem abteilungsinternen „HAINS Airway Kurs“ mitgewirkt [6]. 28,8 % bilden sich häufiger als einmal im Jahr im Bereich Atemwegsmanagement fort.

Kongruent zu bisherigen Weiterbildungsveranstaltungen wünschen sich die ÄrztInnen Lehrveranstaltungen vom Hybrid-Typ (Skill-Trainings mit anschließenden Szenarien/Simulationen, 59,6 % aller Befragten). MitarbeiterInnen, die am neu etablierten Kurs teilgenommen haben, schätzen sich im Vergleich zu ÄrztInnen ohne „HAINS Airway“ signifikant besser ein (U = 222,5, Z = −2,829, *p* = 0,005). Je häufiger ÄrztInnen an einer Fortbildungsveranstaltung zum Thema Atemwegsmanagement teilgenommen haben, desto höher schätzten sie ihre Handlungskompetenz ein (Spearman’s ρ = 0,500, *p* < 0,001).

**Abb. 2:**
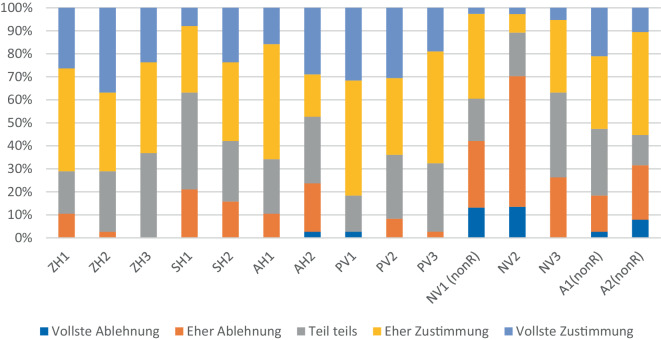
Darstellung der erhobenen Daten mittels Fragebogen zu Transferklima. Fragebogen einsehbar im Zusatzmaterial online. Jedes Item erfragt Faktoren, die eine Übertragung von Erlerntem in die Praxis möglich machen. Die Abkürzungen deuten die jeweiligen Bereiche an, denen die Items zugeordnet werden können: Zielhinweise erhalten (*ZH1–ZH3*), soziale Hinweise erhalten (*SH1* und *SH2*), Aufgabenhinweise erhalten (*AH1* und *AH2*), positive Verstärkung erhalten (*PV1–PV3*), negative Verstärkung erhalten (*NV1–NV3*) und Auslöschung des neu Gelernten gefördert (*A1* und *A2*). Reversed Items sind recodiert worden (*nonR*)

Der Fragebogen zum Transferklima zeigte eine gute Umgebung zur Etablierung neu erlernter Fähigkeiten (Abb. 2). Insbesondere Aspekte der positiven Verstärkung spielen eine wichtige Rolle.

#### Vergleich mit Daten aus 2017

Im Vergleich zu 2017 beteiligten sich mehr jüngere KollegInnen (26 bis 30 Jahre, 11,5 % 2017 und 30,5 % 2023, ohne Signifikanz). Insgesamt konnte hinsichtlich demographischer Parameter der befragten Kollektive kein signifikanter Unterschied dargestellt werden. Prozentual ist der Anteil an erhobenen Koniotomien annähernd idem geblieben (48,9 % aller Fragebogen 2017, 45,8 % aller Fragebogen 2023).

**Abb. 3:**
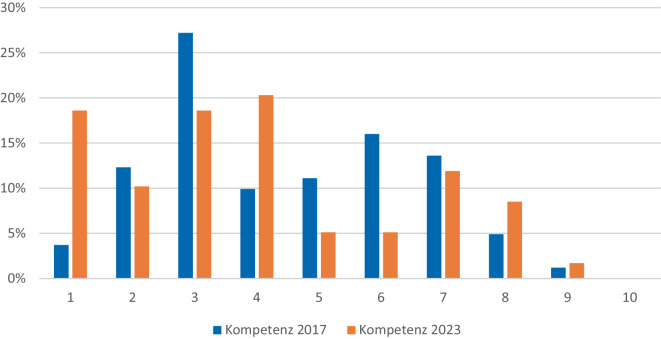
Vergleich der subjektiven Handlungskompetenz zwischen 2017 und 2023. Darstellung der Handlungskompetenz von 1 bis 10 (1 = niedrigste subjektive Handlungskompetenz, 10 = höchste subjektive Handlungskompetenz)

Zwischen den Kohorten aus 2017 und 2023 konnte kein signifikanter Unterschied in der subjektiven Handlungskompetenz gezeigt werden (U = 2078,5, Z = −1,328, *p* = 0,184). Insbesondere mit „1“ haben sich deutlich mehr TeilnehmerInnen 2023 als 2017 eingeschätzt (plus 14,9 %) (Abb. 3), obwohl es zu einem signifikanten Anstieg der Fortbildungsbeteiligung kam (U = 1568,5, Z = −3,369, *p* < 0,001).

### Qualitative Datenerhebung

Jedes Gruppeninterview wurde mit 6 bis 8 Teilnehmenden durchgeführt. Nach jeweils zwei Durchgängen (ÄrztInnen in Weiterbildung, Fach- u. OberärztInnen) konnten die Interview-Reihen aufgrund von inhaltlicher Repetition beendet werden.

65,4 % der Interviewten waren männlich, 34,6 % der TeilnehmerInnen hatten 1 bis 2 Jahre Berufserfahrung, 38,4 % waren über 10 Jahre in der Anästhesiologie. Es ergab sich kein signifikanter Unterschied zwischen der Gruppe der Interviewten und dem Umfrage-Kollektiv hinsichtlich ihres Geschlechtes (χ2(1) = 0,666, *p* = 0,415, φ = −0,088), der Altersverteilung (U = 695,5, Z = −0,713, *p* = 0,476), der Erfahrung (U = 701,5, Z = −0,643, *p* = 0,520) oder des Kompetenzniveau (U = 716,0, Z = −0,544, *p* = 0,586).

Nach Abschluss der Interviews wurden 491 Aussagen der TeilnehmerInnen induktiv zu 13 Themenkomplexen aggregiert.

Die Aussagen überschnitten sich inhaltlich mit den aus den Fragebogen erhobenen Informationen. Dies konnte als Gütekriterium im Rahmen der qualitativen Forschung herangezogen werden („Triangulation“).

## Diskussion

Unsere Studie kann sich auf eine ausgewogene Verteilung im Hinblick auf Geschlecht, Berufserfahrung und Kompetenzniveau stützen, auch die Vergleichbarkeit der drei betrachteten Kohorten ist nach statistischer Analyse gegeben. Der Anteil ärztlicher MitarbeiterInnen mit Koniotomie-Erfahrung entspricht der heterogenen Datenlage (20–31 %) [26, 27]. Es erscheint nachvollziehbar, dass die längere berufliche Tätigkeit die Chance auf eine CICO-Situation erhöht.

### Rolle der Koniotomie

TeilnehmerInnen nehmen die chirurgische Atemwegssicherung als eine zentrale Kompetenz des Atemwegsmanagements und des Fachgebietes Anästhesiologie wahr. Der vom Studienteam eingeführte Begriff der „Kernkompetenz“ wird kritisch betrachtet, da es sich zwar um eine wichtige und notwendige Maßnahme handle, aber eine umfassende Kompetenz aufgrund von Seltenheit, Stressfaktoren und Durchführung bei hohem Handlungsdruck fehle. Die Koniotomie wird klar von der Tracheotomie abgegrenzt, welche von den Befragten in den Bereich der chirurgischen Fachkompetenz eingeordnet wird. Insbesondere die korrekte Verdrängung von Gewebe und Gefäßen wird als Grund beschrieben, der eine chirurgische Grundausbildung verlangt.

Leider konnte durch das umfassende Fortbildungsprogramm keine signifikante Reduktion der Koniotomien nachgewiesen werden. Aussagen hinsichtlich Zu- oder Abnahme der Zahl sind aufgrund der niedrigen Rückläuferquote nur eingeschränkt zu treffen. Eine Verringerung der Koniotomien wäre sicherlich erfreulich, weist aber darauf hin, dass eine vollständige Vermeidung trotz fiberoptischer Intubation, Larynxmaske und Videolaryngoskopie unwahrscheinlich erscheint. Dies wurde auch in den Interviews thematisiert und ist kongruent zur aktuellen Datenlage [8]. Es gilt, das Risiko der chirurgischen Atemwegssicherung trotz optimaler Vorbereitungen in Fortbildungsveranstaltungen immer wieder zu betonen.

Die von der Klinik für Anästhesiologie und Intensivmedizin gelehrte chirurgische Koniotomie mittels Bougie und Skalpell wird als gut erlernbar und übersichtlich beschrieben. Die prozentuale Zunahme chirurgischer Interventionen ist hiermit gut vereinbar.

### Hindernde Faktoren

Im Gesamtkollektiv konnte keine Zunahme der subjektiven Handlungskompetenz gezeigt werden. Im Gegenteil scheint es sogar zu einer Verschlechterung gekommen zu sein. Dies mag durch die Teilnahme vieler KollegInnen des ersten Weiterbildungsjahres und die Einschränkungen bei Fortbildungsveranstaltungen innerhalb der COVID-19-Pandemie bedingt sein. Auch die Seltenheit der chirurgischen Atemwegssicherung spielt wahrscheinlich eine Rolle.

Sicherlich ist die Selbstbeurteilung von der tatsächlichen „handwerklichen“ Fertigkeit abzugrenzen, jedoch zeigen mehrere Studien, dass eine niedrige, subjektive Handlungskompetenz die Gesamtperformance relevant verschlechtert und somit als Erfolgsfaktor relevant bleibt [3, 20].

Innerhalb der Interviews konnten wir als zentralen, hemmenden Faktor zur Durchführung einer Koniotomie die Unsicherheit über einen legitimen Cut-off („Trigger“, einen objektivierbaren, fixierten Wert oder Konstellation) herausarbeiten. Hierbei geht es weniger um eine juristische Absicherung oder Angst vor sozialer Ächtung innerhalb der Klinik, sondern um die Verhältnismäßigkeit der durchgeführten Maßnahme. Wir bewegen uns somit im Formelkreis der „Verantwortlichkeit“ als „kausale Zurechnung begangener Taten“, aber auch Verantwortung durch nicht selbst verantwortete Zuständigkeit [14]. Insbesondere ÄrztInnen in Weiterbildung haben Sorge, dass ihre Unerfahrenheit die erfolgreiche Atemwegssicherung ver- bzw. behindert und erfahrenere KollegInnen eventuell (doch noch) eine Atemwegssicherung erreichen könnten. Die Definition des Cut-off erscheint in den Interviews dementsprechend erstaunlich divers. Es wäre zu evaluieren, ob nicht ein klarerer Cut-off definierbar wäre, um Unsicherheiten und Zeitverzögerung weiter zu reduzieren.

### Blick auf das Atemwegsmanagement und den Algorithmus

Der Atemwegsalgorithmus wurde vielfach als einfach und übersichtlich beschrieben [25]. Dennoch wird dieser nicht aktiv auswendig gelernt, sondern vielmehr durch Praxis erlernt und intuitiv angewandt. Insbesondere in kritischen Situationen beurteilen die TeilnehmerInnen die üblichen Atemwegsalgorithmen [25] als Cognitive Aid für ungeeignet. Dies bedeutet, dass die TeilnehmerInnen in kritischen Situationen tendenziell in der Intuition verharren würden.

Ob eine Umformung der gängigen Atemwegsalgorithmen zu einer suffizienteren Cognitive Aid möglich ist, sollte diskutiert werden. Als positives Beispiel kann hier der mSTaRT-Algorithmus (Vorsichtungsalgorithmus bei Massenanfällen von Verletzten, MANV) benannt werden, der neben der klassischen, algorithmischen Form auch eine Liste kennt [21].

Es wäre zu erwägen, die Anzahl der jeweiligen Versuche deutlicher zu kennzeichnen, ggf. auch die Reihenfolge der Hilfsmittel konkret vorzugeben. Zudem sollte „SpO_2_ ausreichend?“ [21] zugunsten einer konkreten Zahl verändert werden. Analog zu Notfall-Checklisten der Aeronautik sollten die Abwägungszeiten (z. B. zwischen Hilfsmitteln) auf ein Minimum beschränkt werden.

### Weiterbildung und Koniotomie

Insbesondere bei den ÄrztInnen in Weiterbildung herrscht das Bedürfnis vor, den in der S1-Leitlinie dargestellten Atemwegsalgorithmus frühzeitig zu erlernen. Obwohl unsere Studie keine Korrelation zwischen Koniotomie-Zahlen und notfallmedizinischer Tätigkeit nachweisen konnte, sehen ÄrztInnen aller Erfahrungsstufen den Erwerb der Zusatzbezeichnung Notfallmedizin und die damit verbundene Selbstständigkeit als Impulsgeber für ein frühzeitiges Training der chirurgischen Atemwegssicherung. Während kompetente(re) Unterstützung im Setting einer universitären Klinik rasch zur Verfügung steht, wird die Verantwortung in der Präklinik ohne ein „Backup“ mit Sorge betrachtet.

Wie viel Fortbildung aus Sicht der TeilnehmerInnen stattfinden muss, kann aus den Interviews nicht eindeutig herausgearbeitet werden. Die Verbindung aus theoretisch-praktisch orientiertem Training (z. B. Durchführung an Körperspendern) zusammen mit Simulationen mit Fokus auf das Atemwegsmanagement scheint zielführend zu sein. Unser Weiterbildungskonzept korreliert relevant mit der subjektiven Handlungskompetenz. Fortbildung scheint ein die Performance fördernder Faktor zu sein [31]. Nach der Coronapandemie entstand zudem weiterer Fort- und Weiterbildungsbedarf, den es zu adressieren gilt.

## Weitere relevante Themen

Neben Aussagen zur chirurgischen Atemwegssicherung wurden zahlreiche, weitere Themen der anästhesiologischen Praxis beleuchtet. Die Rolle der fiberoptischen Intubation nahm unerwartet viel Raum in den Interviews ein. Insbesondere ÄrztInnen in Weiterbildung beklagen mangelnde, praktische Erfahrung und betonen hieraus entstehende Risiken. Die Videolaryngoskopie wird zwar vielfach als mindestens gleichwertiger Ersatz der Fiberoptik erwähnt, alle TeilnehmerInnen sind sich jedoch einig, dass es weiterhin ausschließliche Indikationen für die fiberoptische Intubation in Spontanatmung gibt. Die Wahl des jeweiligen Verfahrens kann, trotz zahlreicher und immer neuer Prädiktoren für einen schwierigen Atemweg, im klinischen Alltag herausfordernd bleiben.

Der Begriff der Schuld wird mehrfach in den Gesprächen aufgegriffen. Die Abkehr von der fiberoptischen Intubation (FOI) zugunsten der Videolaryngoskopie aufgrund von mangelnder Erfahrung wird als gefährlich und bei kritischen Ereignissen schuldhaft eingestuft. Der Mangel an praktischer Erfahrung stellt ein weltweites Problem dar. Die Einführung von Kursen zur Durchführung der fiberoptischen Intubation, wie sie mittlerweile auch in Deutschland angeboten werden, war daher nur folgerichtig. Trotz Zunahme der Erfahrungen in wach-videolaryngoskopischen Intubationen [12, 29] scheint ein vollständiger Verzicht auf die Prozedur der (wach-)fiberoptischen Intubation zum jetzigen Zeitpunkt eher zur Steigerung des Risikos für CICO-Situationen beizutragen [16].

Auch über die Bedeutung der Anästhesie-Ausweise wurde umfassend diskutiert. Nach flächendeckender Einführung der Videolaryngoskopie erscheint vielen unklar, welche konkreten Indikationen die Ausstellung eines Ausweises noch hat. Vielfach werden Ausweise, die im Rahmen der Prämedikationen mitgebracht werden, eher als unnötige Stigmata empfunden. Soll PatientInnen, die lediglich videolaryngoskopisch intubierbar sind, ein Ausweis ausgestellt werden? Oder lediglich bei Notwendigkeit eines hyperangulierten Spatels? Nur bei der unmöglichen Maskenbeatmung besteht Einigkeit unter den Interviewten.

Ob klinikintern genug Anästhesie-Ausweise ausgestellt werden, wurde ebenfalls diskutiert. Die TeilnehmerInnen betonen, dass häufig Rahmenbedingungen wie die nur eingeschränkt verbesserbare Lagerung (z. B. Einleitung auf dem Herzkatheter-Tisch) oder niedriges Kompetenzniveau als Gründe „vorgeschoben“ werden, keinen Ausweis auszustellen.

Es scheint, dass eine Verbesserung der PatientInnen-Sicherheit erst mit klarer Definition von Ausstellindikationen für die Anästhesie-Ausweise und daraus resultierenden Konsequenzen möglich wird.

## Schlussfolgerung

Die Koniotomie als Ultima Ratio des Atemwegsmanagements ist und bleibt eines der herausforderndsten Szenarien für anästhesiologische Teams. Regelmäßige Fortbildung als Skill-Training mit nachfolgender Einbettung in Simulationsszenarien kann die subjektive Handlungskompetenz signifikant steigern.

Diese Studie konnte zeigen, dass der unklare Cut-off zur Indikation einer sofortigen Koniotomie zur Unsicherheit der anästhesiologischen Teams beiträgt. Dies ist äquivalent zu bisherigen Befragungen. Eine konkretere Formulierung scheint sinnvoll. Diese sollte um eine Cognitive-Aid-Struktur ergänzt werden, die als übersichtlichere Ergänzung der publizierten Atemwegsalgorithmen zu verstehen ist und „notfalltauglich“ angewendet werden kann.

Die chirurgische Atemwegssicherung ist als Teil des Atemwegsmanagements eingebettet in ein umfangreiches Sicherheitsnetz, welches auch die ausreichende Praxis der fiberoptischen Intubation und eine korrekte Evaluation prä- und postanästhesiologisch miteinbezieht. Der Anästhesie-Ausweis kann hierbei ein nützliches, die PatientInnen-Sicherheit erhöhendes Tool sein, sofern angepasste Definitionen und Anwendungen festgelegt werden.

## Limitationen

Eine höhere Rückläuferquote hätte die Qualität der hier präsentierten Daten verbessert. Es ist fraglich, ob eine fehlende Teilnahme an der Studie auf keine Erfahrung mit der chirurgischen Atemwegssicherung hindeutet oder sonstige, andere Gründe vorlagen.

Einzelne Teilnehmerinnen haben aufgrund ihrer langjährigen beruflichen Tätigkeit an beiden Umfragen teilgenommen. Eine pseudonymisierte, nachträgliche Paarung mit Ergebnissen von 2017 konnte nicht gelingen. Die Anzahl an Koniotomien zu den zwei Befragungszeitpunkten kann somit anteilig, identische Ereignisse darstellen.

Die Fokussierung von Umfragen und Interviews auf eine einzelne Klinik kann einen relevanten Bias darstellen. Der Abgleich mit anderen Kliniken für Anästhesiologie (mit und ohne differenzierten Weiterbildungskonzepten im Bereich Atemwegsmanagement) erscheint als sinnvolle Fortführung der hier dargestellten Studie.

Die seit vielen Jahren etablierte, qualitative Methodik im Spannungsfeld wissenschaftstheoretischer Erwägungen ist abzugrenzen von klassischer, quantitativer Forschung. Die hier dargestellten Aussagen sowie Generalisationen unterliegen möglichen Unschärfen im Rahmen der Interviews und des Extraktionsprozesses und müssen stets angemessen kontextualisiert werden. Qualitative Methodik dient nicht der Signifikanzmessung, sondern als „Fenster“ in die individuelle Realität der Interviewten und kann so als Impulsgeber für weitere Forschungs- und Weiterbildungskonzepte dienen.

## Fazit für die Praxis

- Die Situation des „cannot intubate – cannot oxygenate“ gehört weiterhin zu den herausforderndsten Momenten während der anästhesiologischen Tätigkeit.
- Die Videolaryngoskopie hat im mentalen Modell des Anästhesie-Personals deutlich an Bedeutung hinzugewonnen, teilweise zu Ungunsten der wachfiberoptischen Intubation.
- Die Rolle des Anästhesie-Ausweises sollte im Hinblick auf die flächendeckende Einführung der Videolaryngoskopie neu definiert und differenziert werden.
- Die Unsicherheit über einen sinnvollen „Cut-off“ zur Indikation der sofortigen Koniotomie konnte als zentraler Punkt für die geringe subjektive Handlungskompetenz ausgemacht werden.
- Regelmäßiges Skill-Training und Beübung an Simulationsszenarien stellen ein sinnvolles Weiterbildungskonzept für das Erlernen der chirurgischen Atemwegssicherung dar.
- Die Entwicklung einer „notfalltauglichen“ Cognitive-Aid-Struktur, in Synergie zu unseren aktuell geltenden Atemwegsalgorithmen, erscheint aufgrund der hohen Belastung in CICO-Situationen sinnvoll.

## Supplementary Information

ESM1: Zusatzmaterial 1

## Data Availability

Auf Nachfrage sind sämtliche erhobenen Daten, die diese Publikation betreffen, bei dem korrespondierenden Autor erhältlich.
