# Supplementary material for: The mindset of anesthesiologists while performing surgical airway management
Source: Anaesthesiologie. 2026 Jun 2;75(8):579–88. [Article in German] doi: 10.1007/s00101-026-01696-w (PMC13427881; doi:10.1007/s00101-026-01696-w)
Supplement: Supplementary file 1 — ESM1: Zusatzmaterial 1 [file 101_2026_1696_MOESM1_ESM.pdf]

Fragebogen Teil 1

Demographische Daten

**Alter (Jahre)**	20-25 O	26-30 O	31-40 O	41-50 O	>50 Jahre O
**Geschlecht**	M O		W O		D O
**Dauer der Tätigkeit als ÄrztIn in der Anästhesie (Jahre)**	1-2 O	3-4 O	5-10 O	11-20 O	>20 O
**Berufsbezeichnung**	ÄiW O		FÄ O		OÄ oder CÄ O
**Zusatzbezeichnung Notfallmedizin (oder Äquivalent)**	O Ja			O Nein	

Erfahrungen mit der chirurgischen Atemwegssicherung

Wie oft haben Sie schon eine chirurgische Atemwegssicherung (Koniotomie) am Patienten durchgeführt?

**Noch nie**	**1-mal**	**2-mal**	**3-mal**	**4-mal**	**5-mal oder mehr**
O	O	O	O	O	O

In welchem Setting haben Sie die chirurgische Atemwegssicherung durchgeführt (Mehrfachantworten möglich)**?**

**Rettungsdienst**	**ZNA**	**OP/Einleitung**	**ITS**	**Sonstiges**
O	O	O	O	O

Welches Verfahren haben Sie bei Ihrer chirurgischen Atemwegssicherung (Koniotomie) bisher eingesetzt (Mehrfachantworten möglich)**?**

**Direktpunktion (dünnlumige Kanüle)**	**Direktpunktion (weitlumige Kanüle)**	**Kanüle in Seldingertechnik**	**Offen (Skalpell + Spekulum)**	**Offen (Skalpell + Bougie)**	**Sonstiges**
O	O	O	O	O	O

Wie schätzen Sie Ihre Handlungskompetenz im Rahmen einer chirurgischen Atemwegssicherung (Koniotomie) zurzeit ein? (1 = unsicher, 10 = sicher)

**1**	**2**	**3**	**4**	**5**	**6**	**7**	**8**	**9**	**10**

Wie viele Trainingsmöglichkeiten der chirurgischen Atemwegsicherung nutzen Sie pro Jahr?

[ ] **Mal pro Jahr**

Wie stellen Sie sich ein optimales Training für die chirurgische Atemwegssicherung vor?

**Vorlesung (theoretische Wissensvermittlung)**	**Skilltraining (Training am Modell)**	**Simulationstraining (Szenarien im Team)**	**Hybrid-Training (Skilltraining + Simulation)**	**eLearning (Online-basiertes Eigenstudium)**	**Sonstiges**
O	O	O	O	O	O

Haben Sie bereits an einem HAINS Airway Simulationskurs mit dem Schwerpunkt auf Atemwegmanagement teilgenommen?

O **Ja** O **Nein**

Bitte wenden!

Fragebogen zum Transferklima *(nur wenn bereits an HAINS Airway Simulationskurs teilgenommen)*

*Für die Beantwortung dieser Fragen denken Sie bitte an Ihre täglichen **Arbeit(-serfahrungen)** nach der Teilnahme an dem HAINS Airway Training.*

Frage	Vollste Ablehnung	Eher Ablehnung	Teils teils	Eher Zustimmung	Vollste Zustimmung
Meine Vorgesetzten setzen Ziele, welche mich bestärken die im Training erworbenen Kenntnisse anzuwenden.	O	O	O	O	O
Meine Vorgesetzten erwarten von mir, dass ich die Inhalte aus dem Training im beruflichen Alltag anwende.	O	O	O	O	O
Meine KollegInnen erwarten von mir, dass ich meine Tätigkeiten gemäß den Inhalten des Trainings ausführe.	O	O	O	O	O
Meine KollegInnen können mir zeigen, wie ich die Inhalte des Trainings in der Arbeit anwenden kann.	O	O	O	O	O
Meine Vorgesetzten geben mir die Möglichkeit, die Inhalte des Trainings in der täglichen Arbeit anzuwenden.	O	O	O	O	O
In der Arbeit habe ich die Möglichkeiten mich so zu verhalten, wie ich es im Training gelernt habe.	O	O	O	O	O
Es sind ausreichend Ressourcen (Personen o. Equipment o. Zeit) vorhanden, um die Arbeit so zu erledigen, wie es in den Trainings vermittelt wurde.	O	O	O	O	O
Wenn ich die Inhalte der Trainings anwende, wird die Arbeit leichter.	O	O	O	O	O
Meine Vorgesetzten erkennen es positiv an, wenn ich die Inhalte der Trainings korrekt umsetze.	O	O	O	O	O
Meine KollegInnen merken es positiv an, wenn ich meine Arbeit so ausführe, wie in den Trainings gelernt.	O	O	O	O	O
(Erfahrenere) KollegInnen machen sich schon mal über die in den Trainings vermittelten Inhalten lustig.	O	O	O	O	O
Wenn ich die Inhalte der Trainings nicht anwende, werde ich ermahnt.	O	O	O	O	O
Um meine Arbeit richtig auszuführen, muss ich mich genau an die Techniken und Verfahrensweisen halten, die ich in den Trainings erlernt habe.	O	O	O	O	O
Für meine Vorgesetzten ist es eher nebensächlich, ob ich die Inhalte der Trainings anwende, solange die Arbeit erledigt wird.	O	O	O	O	O
Ich habe wenige Gelegenheiten die Inhalte der Trainings anzuwenden, so dass ich diese nicht vollständig verinnerlichen kann.	O	O	O	O	O

Demographische Daten

**Alter (Jahre)**	20-25<br>O	26-30<br>O	31-40<br>O	41-50<br>O	>50 Jahre<br>O
**Geschlecht**	M<br>O		W<br>O		D<br>O
**Dauer der Tätigkeit als ÄrztIn in der Anästhesie (Jahre)**	1-2<br>O	3-4<br>O	5-10<br>O	11-20<br>O	>20<br>O
**Berufsbezeichnung**	ÄiW<br>O		FÄ<br>O		OÄ oder CÄ<br>O
**Zusatzbezeichnung Notfallmedizin (oder Äquivalent)**	O<br>Ja			O<br>Nein	

Erfahrungen mit der chirurgischen Atemwegssicherung

Wie oft haben Sie schon eine chirurgische Atemwegssicherung (Koniotomie) am Patienten durchgeführt?

Noch nie	1-mal	2-mal	3-mal	4-mal	5-mal oder mehr
O	O	O	O	O	O

Zusatzmaterial zum Beitrag „Das „Mindset“ bei der Durchführung einer chirurgischen Atemwegssicherung durch AnästhesistInnen“ von Jan Carlo Del Tedesco, Lion Sieg, Markus Flentje et al. (2026) in *Die Anaesthesiologie.*
Beitrag und Zusatzmaterial stehen Ihnen auf www.springermedizin.de zur Verfügung. Bitte geben Sie dort den Beitragstitel in die Suche ein.
